# A 4-month-old baby presenting with dermal necrotizing granulomatous giant cell reaction at the injection site of 13-valent pneumococcal conjugate vaccine: a case report

**DOI:** 10.1186/1752-1947-8-285

**Published:** 2014-08-24

**Authors:** Ahmed R Alsuwaidi, Alia Albawardi, Navidul Haq Khan, Abdul-Kader Souid

**Affiliations:** 1 Department of Pediatrics, United Arab Emirates University, P.O. Box 17666, Al-Ain, UAE; 2 Department of Pathology, United Arab Emirates University, P.O. Box 17666, Al-Ain, UAE; 3 Clinical Laboratory, Al Ain Hospital, Al-Ain, UAE

**Keywords:** Adjuvants, Adverse events, Necrotizing granulomatous giant cell reaction, Pneumococcal conjugate vaccine

## Abstract

**Introduction:**

Adjuvants (for example, aluminum salts) are frequently incorporated in licensed vaccines to enhance the host immune response. Such vaccines include the pneumococcal conjugate, combinations of diphtheria–tetanus/acellular pertussis, tetanus– diphtheria/acellular pertussis, hepatitis B, some *Haemophilus influenzae* type b, hepatitis A, and human papillomavirus. These preparations have been associated with complicated local adverse events, especially if administered subcutaneously or intradermally in comparison to deep intramuscular injection. We describe a severe inflammatory reaction at the site of an injection of 13-valent pneumococcal conjugate vaccine.

**Case presentation:**

A 4-month-old Arab baby boy developed dermal necrotizing granulomatous giant cell reaction at the injection site (right anterior thigh) of the second dose of 13-valent pneumococcal conjugate vaccine. Ziehl–Neelsen and periodic-acid Schiff were negative. This reaction probably resulted from improper intramuscular administration because the first (at 2 months of age) and third (at 10 months of age) doses were uneventful.

**Conclusions:**

Dermal necrotizing granulomatous reactions are a serious complication of the 13-valent pneumococcal conjugate vaccine. Health care providers need to administer this preparation deeply into a muscle mass. Completing the vaccine series is an acceptable option. Physicians are encouraged to report their experience with completing vaccine series following adverse events.

## Introduction

Introduction of the 7-valent pneumococcal conjugate vaccine (PCV7) has resulted in a substantial drop in the rate of invasive pneumococcal disease. However, non-vaccine serotypes have emerged. The 13-valent pneumococcal conjugate vaccine (PCV13) was subsequently developed and is currently used in many countries [1].

The safety profile of PCV13 is similar to that of PCV7. Local reactions are common including tenderness (47%), swelling (29%), and redness (36%). These adverse events are usually mild and occur after any dose of the infant series [2].

We describe a severe dermal inflammatory reaction at the site of the injection of the second PCV13 dose. The report aims at improving vaccination safety.

## Case presentation

**Figure 1:**
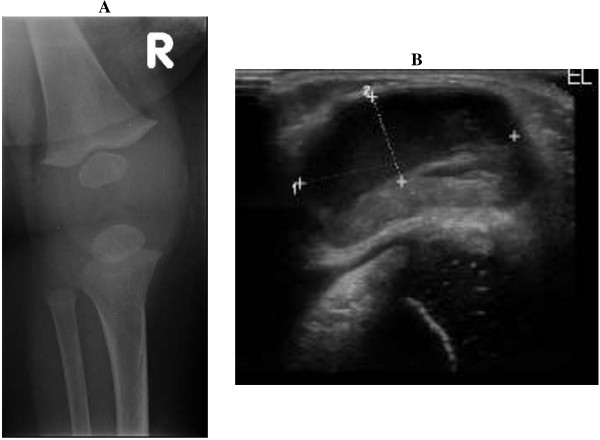
**Radiological images of the lesion 3 weeks after administering the 13-valent pneumococcal conjugate vaccine.***Panel****A****:* Right knee X-ray showing anterior fluid collection. *Panel****B****:* Ultrasound of the right knee showing swelling (measuring 2.9cm transversely and 1.5cm anteroposteriorly) of the anterior aspect of the knee due to fluid collection.

A 4-month-old previously healthy Arab baby boy presented with progressive swelling, redness, and firmness at the injection site (right anterior thigh) of his second PCV13 dose. The findings were noticeable within 24 hours of the vaccination and progressed rapidly into the prepatellar area.At 3 weeks, the clinical findings in the anterior aspect of his knee were very prominent (Figure 1), prompting draining of the lesion. The aspirate (10mL) yielded a sterile pus (Gram stain and bacterial culture were negative). Nevertheless, he received an empiric course of flucloxacillin.

**Figure 2:**
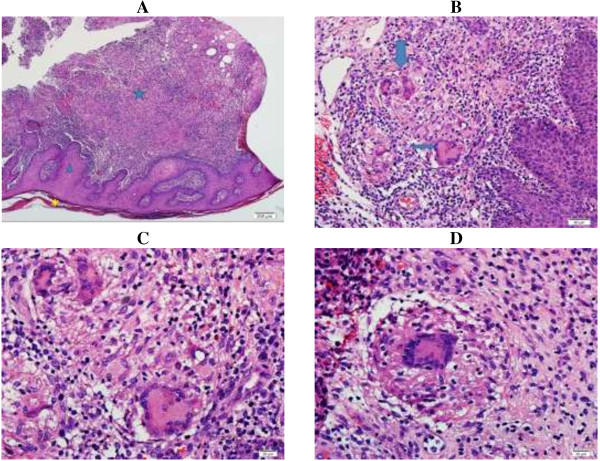
**Histopathology. ***Panel****A****:* Low power view (4×): Tissue section of skin demonstrates hyperkeratosis (*), epidermal acanthosis (thickening, triangle) and an inflammatory dermal process (star). *Panel****B****:* Intermediate power view (20×): Dermal granuloma formation accompanied by multinucleated giant cells (arrows). *Panels****C***-***D***: High power view (40×): Granulomatous giant cell inflammatory reaction, collection of epithelioid histiocytes admixed with lymphocytes rimmed by multinucleated giant cells.

At 8 weeks, he had a second drainage and open skin and soft tissue biopsies. The findings revealed erythema induratum, a granulomatous inflammation with fibrinoid and caseous necrosis (Figure 2). Ziehl–Neelsen and periodic-acid Schiff stains (special stains for mycobacteria and fungi) were negative. Gram stain showed 3+ pus cells with no organisms. Wound bacterial culture grew 1+ *Klebsiella pneumoniae*, considered a secondary infection. The patient received oral cefixime.

At 10 weeks, the area appeared better; the swelling and other clinical signs were regressing. He received the third PCV13 dose at 10 months of age and the injection was uneventful.

## Discussion

The precise mechanism of this prolonged and deleterious inflammatory reaction to PCV13 remains obscure. Adjuvants, such as aluminum salts, are frequently incorporated in licensed vaccines (including PCV13) to enhance the host immune response [3]. Local reactions (for example, granuloma formation) induced by these adjuvants are relatively common [4].

Bordet *et al*. have reported cases of subcutaneous nodules with a necrotizing granulomatous reaction at the site of a previous injection of the aluminum hydroxide-containing vaccine, Tetracoq® (tetanus, diphtheria, *Bordetella pertussis*, poliovirus) [5]. Several cases of persistent itchy subcutaneous nodules (lasting for years) and hypersensitivity to aluminum after diphtheria–tetanus/acellular pertussis/polio+Hib vaccination have been also described [6]. Consistently, one study in pigs demonstrated that aluminum hydroxide could invoke a granulomatous reaction [7].

The American Academy of Pediatrics recommends that administration of vaccines containing adjuvants (for example, aluminum present in vaccines recommended for intramuscular injection including PCV13) should be deep into a muscle mass. Subcutaneous or intradermal injections of these preparations are associated with an increased incidence of local irritation, inflammation, granuloma formation, and tissue necrosis [8].

This baby had no adverse events following the first and third PCV13 doses. Thus, it is unlikely that the observed response to the second PCV13 dose was an allergic reaction. It is probable that an improper technique of administration was responsible for this reaction. Proving this possibility retrospectively, however, is difficult.

The specific stains and cultures did not support mycobacterial or fungal infection. The positive wound bacterial culture was most probably a secondary hospital-acquired infection and managed with a proper antibiotic. The initial antibiotic course, however, was empiric for a suspected cellulitis.

## Conclusions

Dermal necrotizing granulomatous giant cell reaction is a serious complication of the 13-valent pneumococcal conjugate vaccine. These lesions require only conservative treatment. Babies may react to any dose of the vaccine series. Physicians are encouraged to report their experience with completing vaccine series following adverse events. Appropriate administration technique of adjuvant-containing vaccines is crucial.

## Consent

Written informed consent was obtained from the patient’s parent for publication of this case report and accompanying images. A copy of the written consent is available for review by the Editor-in-Chief of this journal.

## Competing interests

The authors declare that they have no competing interests.

## Authors’ contributions

ARA and AKS are the managing pediatricians; they drafted the manuscript. AA and NHK are the pathologists who interpreted the histology. All authors have approved the final manuscript.
